# Integrative Physiotherapy Management of Cervical Radiculopathy and Concurrent Tarlov Cysts

**DOI:** 10.7759/cureus.57204

**Published:** 2024-03-29

**Authors:** Harsh R Nathani, Vrushali Athawale, Grisha Ratnani

**Affiliations:** 1 Community Health Physiotherapy, Ravi Nair Physiotherapy College, Datta Meghe Institute of Higher Education and Research, Wardha, IND

**Keywords:** manual muscle testing, myelography, cervical radiculopathy, perineural cysts, tarlov cysts

## Abstract

Tarlov cysts, also known as perineural cysts, are usually associated with lumbar discomfort and neurological deficits, with an uncertain etiology that may involve genetic predisposition and collagen disorders, possibly influenced by traumatic events and hemorrhagic episodes. Diagnostic methods such as magnetic resonance imaging or computed tomography myelography are commonly employed and treatment approaches range from conservative measures to more invasive interventions. This case involves a 42-year-old female with cervical pain and upper limb symptoms; a comprehensive assessment, including diagnostic imaging and physiotherapeutic interventions, resulted in significant improvements in pain intensity, range of motion, manual muscle testing, and functional scale scores after a two-week physiotherapy intervention. These findings contribute to advancing our understanding of managing Tarlov cysts associated with cervical radiculopathy, highlighting the potential efficacy of physiotherapeutic interventions in enhancing patient outcomes.

## Introduction

Tarlov cysts, or perineural cysts, primarily reside within the interstitial space surrounding the perineurium and endoneurium layers encapsulating the nerve fibers [1]. Predominantly located along sacral nerve roots, particularly at the S2 level, these cysts accumulate cerebrospinal fluid (CSF) and, in certain instances, contain blood due to hemorrhagic occurrences. Despite often being asymptomatic, symptomatic cases may manifest as persistent lumbar discomfort [2]. In more severe scenarios, Tarlov cysts can precipitate radiculopathy and leg weakness, as well as urinary, digestive, and sexual dysfunction [3]. The cause of Tarlov cysts remains unknown, but they are recognized for their expansion brought on by the input of CSF fluid via a vent network and their resistance to self-reduction. The arachnoid mater frequently exhibits circumferential bruising near the cyst's entry, essential for valve development [4]. There may be a connection between Tarlov cysts and collagen diseases such as Ehlers-Danlos syndrome and Marfan syndrome [5]. The pathophysiological mechanism of Tarlov cysts involves trauma, hemorrhage, congenital arachnoidal proliferation, and genetic risk factors, which enhance sacral nerve roots and promote cyst formation. Tarlov cysts have a one-way valve mechanism that permits growth but restricts size reduction, connecting them to the subarachnoid space [6].

Certain Tarlov cysts show symptoms, worsening over time and potentially leading to neurological issues. They can cause discomfort by irritating the periosteum, eroding nearby sacral bone, and triggering fibrotic alterations observed upon histopathological investigation. Internal bleeding within a cyst may lead to an inflammatory reaction, resulting in hemosiderin deposition [7]. Tarlov cysts can have many locations or just one location, with symptoms including radicular discomfort, dermal pain, decreased sensation, or weakness. Perineal discomfort, bowel and bladder problems, and sexual dysfunction are common among individuals with Tarlov cysts [8]. An extensive neurological evaluation is essential for diagnosing and treating Tarlov cysts. Patient evaluation, particularly when treating Tarlov cysts or cauda equina syndrome, is critical in healthcare [9]. Magnetic resonance imaging (MRI) is the preferred assessment method, with computed tomography (CT) myelography used when MRI is not appropriate [10].

Treatment options for Tarlov cysts include conservative, medicinal, and invasive procedures. Conservative care is preferred for asymptomatic cysts, while symptomatic cysts may require medical therapy employing pharmaceutical drugs. Caudal epidural steroid injections are used for bladder pain syndrome [11]. Percutaneous therapy options include cyst aspiration, fibrin glue injection, and open surgical methods, with decompressive laminectomy considered for cysts larger than 1.5 cm. Shunting operations have risks and side effects to consider, with treatment choices tailored to the patient's needs, preferences, and the medical professional's experience [12]. Physiotherapy plays a crucial role in managing minimally affected patients with Tarlov cysts, especially post-operatively. Physical therapists prescribe various interventions like prone lumbar extension mobilization, hip strengthening exercises, and other neurodynamic management strategies. After two months of nonsurgical management, patients often experience resolution of pain and decreased neurogenic symptoms, highlighting the effectiveness of physiotherapy [13].

## Case presentation

Patient information

A 42-year-old female patient presented to the neurology outpatient department (OPD) with a recent history of cervical pain radiating to bilateral upper limbs, cervicogenic headache, and occasional tinnitus. The onset of symptoms followed a road traffic accident a month prior, during which she experienced a fall over her back. Initial conservative management included rest and the application of a cervical collar and lumbosacral belt. However, a week later, she began experiencing neck pain; initially, it was of minimal intensity, which progressively intensified and extended to involve both the upper limbs. Subsequently, the patient underwent MRI, received pharmacological intervention, and was recommended for physiotherapeutic intervention for further management.

Clinical findings

Upon following the patient's verbal consent, a comprehensive assessment was conducted. The female patient was an ectomorphic build. The patient was conscious, cooperative, and well-oriented to time, place, and person. Her pain was assessed on the visual analogue scale (VAS) which was 4/10 on rest and 7/10 on activity and was insidious in onset, which was aggravated on neck flexion, and was relieved on rest and the medication site was the back of the neck and bilateral upper limbs and was sharp shooting in nature. Vital signs were stable, with both shoulders demonstrating slight elevation. The patient utilized a soft cervical collar, and a forward head posture was observed. Grade 2 tenderness was noted over the lateral aspect of both arms and spasm was present over trapezius muscle belly. Movement quality in the cervical region was characterized by pain and incompleteness. The end feel was firm, and bilateral tightness of the upper trapezius was identified upon examination. Manual muscle testing (MMT) and range of motion (ROM) assessment for the cervical spine are shown in Table 1 and Table 2. Special tests done for the examination are depicted in Table 3.

**Table 1:** MMT charting for cervical muscles according to MRC grading MMT: Manual muscle testing; Grade 3: Active movement against gravity; Grade 4: Active movement against gravity and resistance; Grade 5: Normal power

Muscles	Right	Left
Cervical flexors	3/5	3/5
Cervical extensors	3/5	3/5
Lateral flexors	3/5	3/5
Cervical rotators	4/5	3/5

**Table 2:** Physiotherapeutic assessment of cervical ROM using a goniometer ROM: Range of motion Reduction in ranges of cervical spine as compared to normal

Movements	Patient's range	Normal ranges
Cervical flexion	0-25°	0-45°
Cervical extension	0-30°	0-45°
Cervical lateral flexion (right)	0-30°	0-45°
Cervical lateral flexion (left)	0-25°	0-45°
Cervical rotation (right)	0-45°	0-60°
Cervical rotation (left)	0-40°	0-60°

**Table 3:** Assessment of cervical spine via special test for diagnosing nerve root involvement ULTT 1: Upper limb nerve tension test (median nerve); ULTT 2: Upper limb nerve tension test (radial nerve); ULTT 3: Upper limb nerve tension test (ulnar nerve)

Special tests	Result
Cervical foraminal compression test	Positive
Cervical distraction test	Positive
ULTT 1, ULTT 2, ULTT 3	Positive

Diagnostic assessment

**Figure 1:**
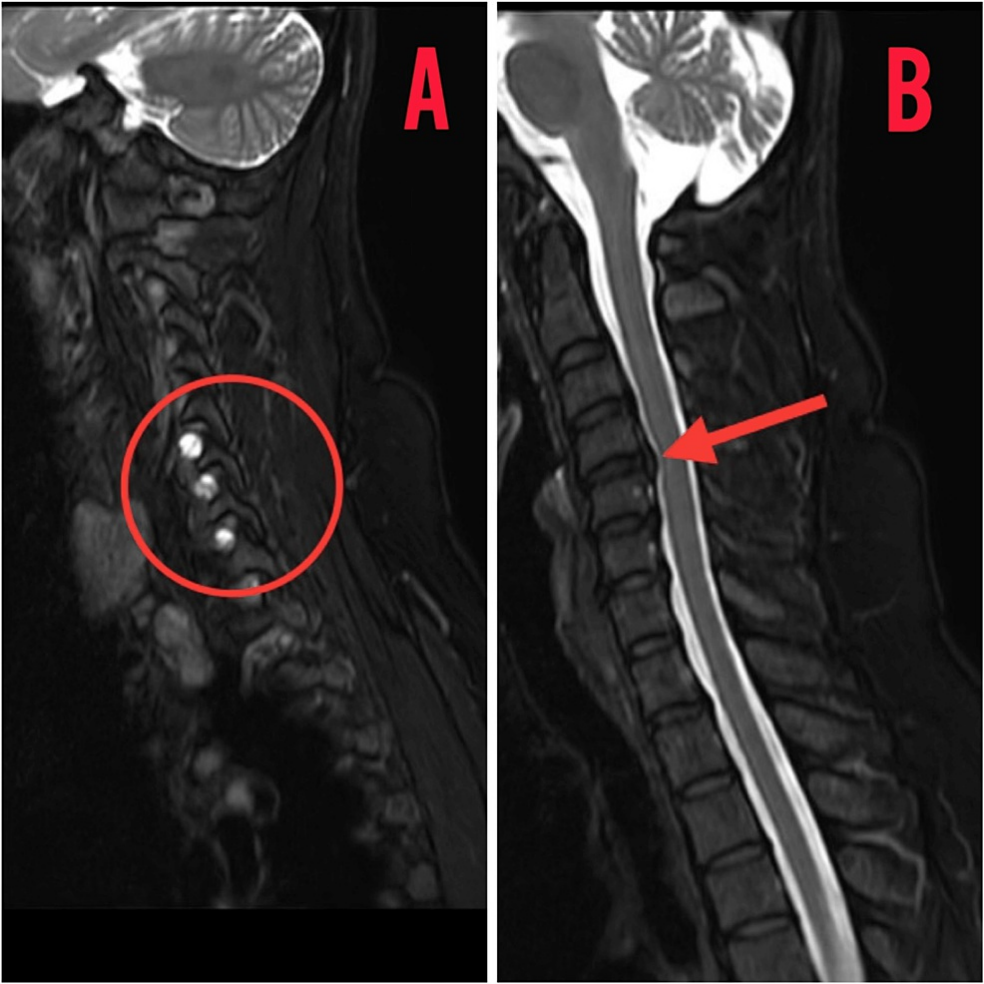
MRI of the cervical spine in the sagittal view showing hyperintense shadows of cyst and bulging of discs MRI: Magnetic resonance imaging (A) Hyperintense lesions over C4,C5,C6 vertebrae; (B) disc bulging over C3-C4, C4-C5, C5-C6, C6-C7 vertebrae levels

**Figure 2:**
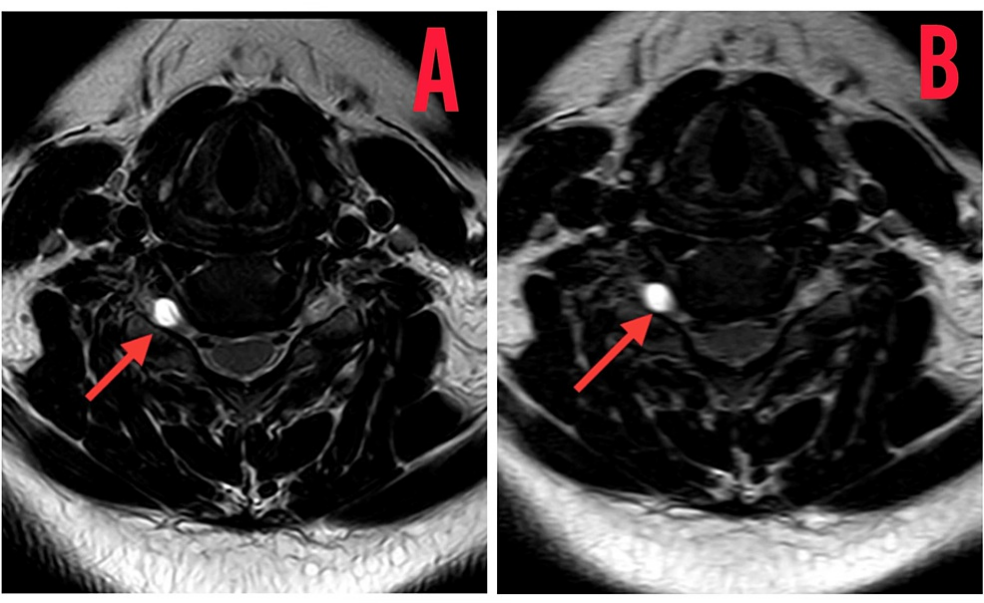
MRI of the cervical spine transverse view depicting a Tarlov cyst 5.91 mm in size MRI: Magnetic resonance imaging (A0 Tarlov cyst shadow in the transverse view; (B) hyperintense lesion of the Tarlov cyst

The patient underwent a diagnostic assessment, including an MRI scan of the entire spine, which revealed disc bulges at the L2-L3, L3-L4, and L4-L5 segments. Furthermore, an MRI of the cervical spine exhibited extraneous disc bulges at the C3-C4, C4-C5, C5-C6, and C6-C7 levels, resulting in the straightening of the cervical spine as shown in Figure 1. Notably, hyperintense density lesions were identified over the C4, C5, C6, and C7 vertebrae during the cervical spine MRI assessment as shown in Figure 2.

Therapeutic interventions

**Figure 3:**
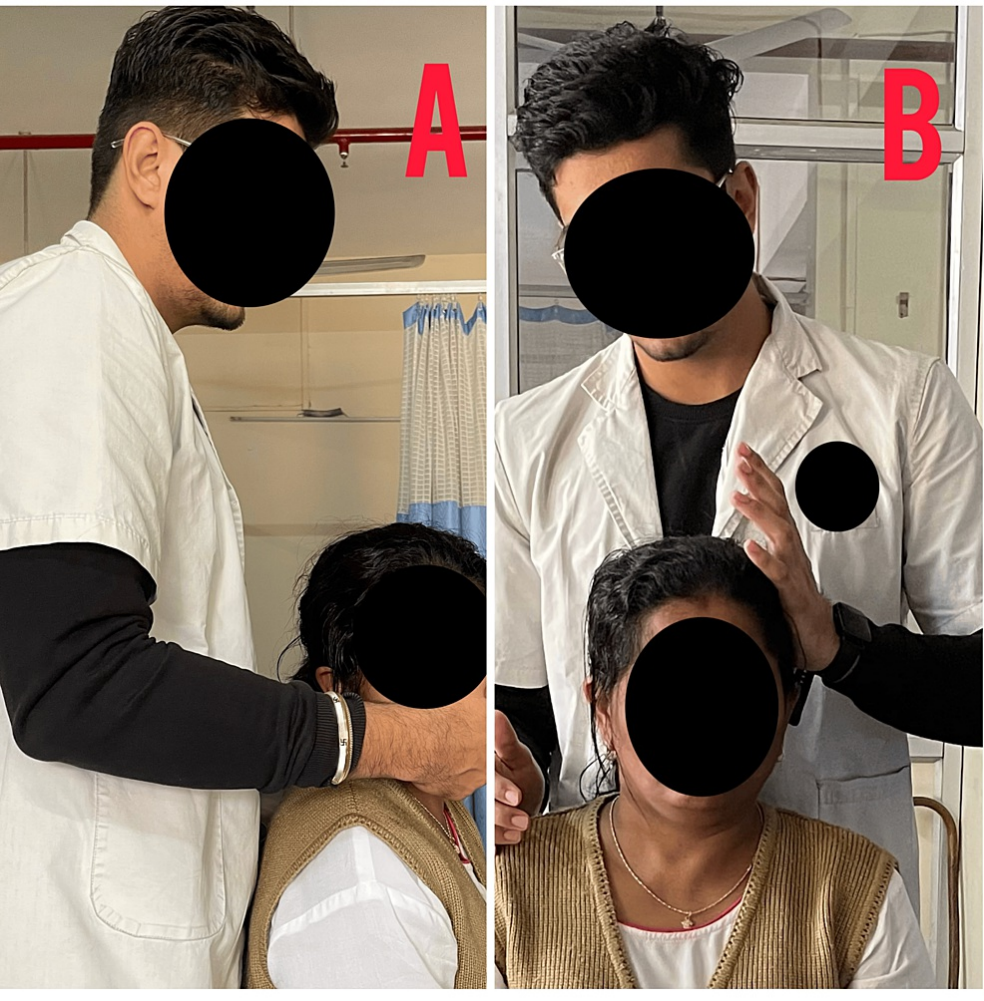
PROM exercises and isometrics for cervical muscles PROM: Passive range of motion (A) Passive range of motion exercise for cervical musculature; (B) isometrics for cervical musculature

**Figure 4:**
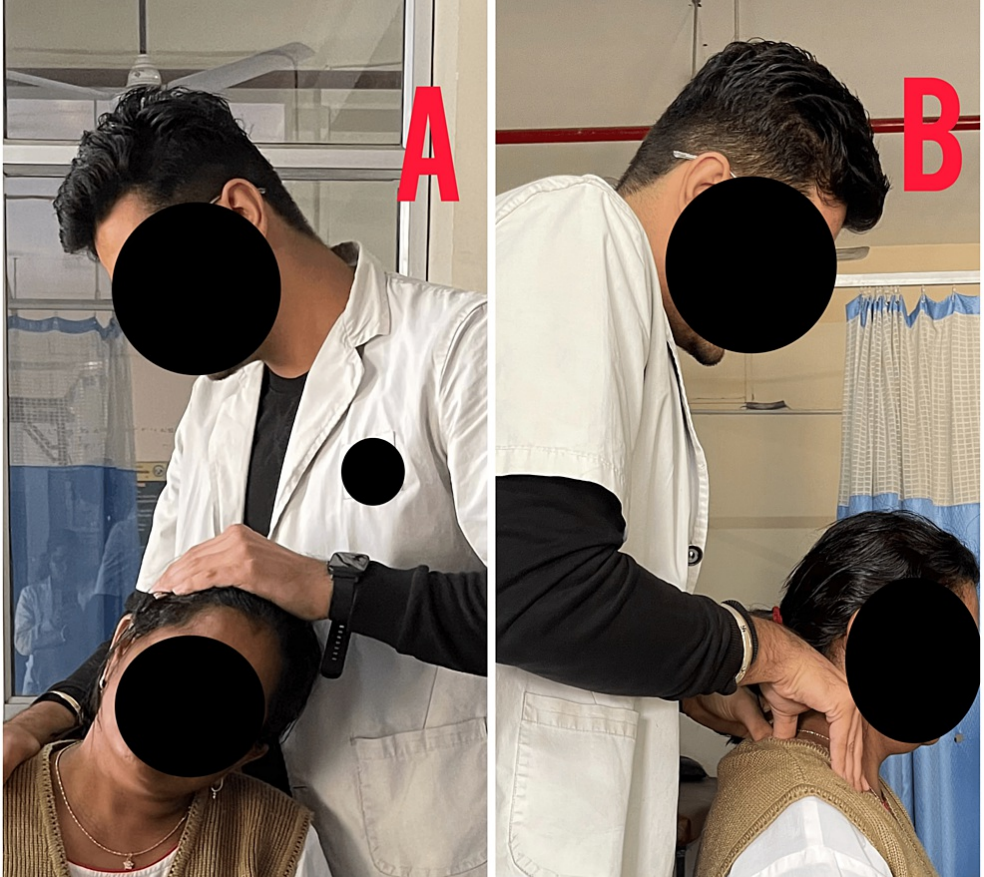
Stretching of trapezius and soft tissue MFR MFR: Myofascial release (A) Stretching of trapezius muscle; (B) myofascial soft tissue release for trapezius muscle

**Figure 5:**
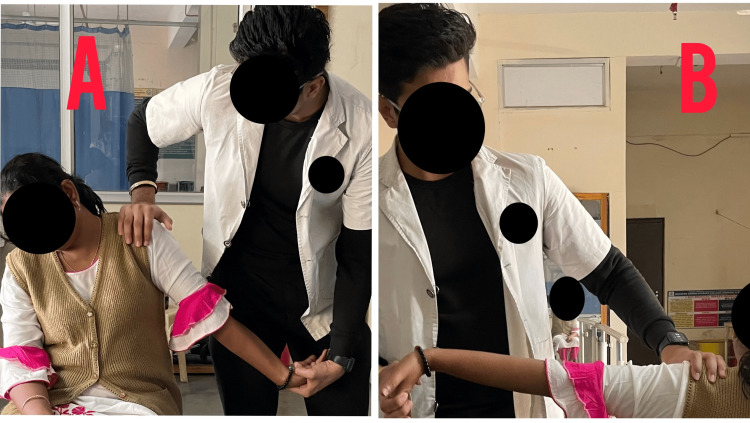
Neural glides maneuver for radial and median nerve (A) Radial nerve sensitizing gliding maneuver; (B) median nerve sensitizing gliding maneuver

Table 4 depicts the intervention provided to the patient, Figure 3 shows the passive range of motion exercises, Figure 4 shows the stretching of cervical muscles, and Figure 5 shows the nerve sliders given to the patient.

**Table 4:** Physiotherapy rehabilitation incorporated for the patient TENS: Transcutaneous electrical nerve stimulation

Sr. No.	Goals	Physiotherapeutic approaches	Rationale
1.	To alleviate pain and discomfort	TENS application over the cervical area	TENS works by delivering low-frequency electrical currents to the skin, which can modulate pain transmission by activating A-beta fibers
2.	To reduce impingement of the nerves	Cervical traction via traction table	It serves as a valuable tool in reducing compressions on neural structure
3.	To improve cervical spinal mobility	Active and passive range of motion exercises for the cervical spine	Enhances cervical movement and reduces stiffness in the neck
4.	To improve muscle strength	Isometrics for cervical flexors extensors and shoulder isometrics for flexion extension abduction and adduction	Provides stability and supports the cervical spine
5.	To minimize and resolve cervical straightening	Chin tucks and scapular retraction exercises	Reduces strain on cervical spine and prevents worsening of the symptoms
6.	To prevent muscle tightness and muscle spasm	Stretching for trapezius muscle and soft tissue myofascial release for upper trapezius fibers	Improves flexibility of joints and muscles
7.	To improve neural mobility and nerve related symptoms	Neurodynamic exercises by neural glides for radial, ulnar and median nerve	Improves nerve mobility and mitigates neural tension

Outcome measures

Table 5 shows the outcomes of the intervention for the patient.

**Table 5:** Outcome measure scale utilized for the patient to evaluate the progression of the patient VAS: Visual analogue scale; ROM: range of motion; MMT: manual muscle testing; PSFS: patient-specific functional scale GROC- Global rating of change MMT- Grade 3- Active movement against gravity Grade 4- Active movement against gravity and resistance Grade 5- Normal movement PSFS- 0- Unable to perform activity 10- Able to perform activity at the same level as before injury or problem GROC- +3- Somewhat better +5- Quite a bit better +7- A very great deal better

Outcomes	Day 1 assessment		End of Week 1		End of Week 2	
VAS	On rest- 4/10		On rest- 2/10		On rest- 1/10	
	On activity- 7/10		On activity- 5/10		On activity- 2/10	
ROM						
Cervical flexion	0-25°		0-35°		0-40°	
Cervical extension	0-30°		0-40°		0-45°	
Cervical lateral flexion (right)	0-30°		0-40°		0-45°	
Cervical lateral flexion (left)	0-25°		0-40°		0-45°	
Cervical rotation (right)	0-45°		0-50°		0-55°	
Cervical rotation (left)	0-40°		0-50°		0-55°	
MMT	Right	Left	Right	Left	Right	Left
Cervical flexors	3/5	3/5	4/5	4/5	4/5	4/5
Cervical extensors	3/5	3/5	4/5	4/5	5/5	5/5
Lateral flexors	3/5	3/5	4/5	4/5	5/5	4/5
Cervical rotators	4/5	3/5	4/5	4/5	5/5	5/5
PSFS	4/10		6/10		10/10	
GROC	+3/+7		+5/+7		+7/+7	

## Discussion

Tarlov cysts, an uncommon cervical and lumbar discomfort that primarily affects women, can produce localized pain and neurological pathways [14]. Symptoms may include cervical radiculopathy, tingling, numbness, sciatica, sacral discomfort, vaginal paraesthesia, sensory abnormalities, and bladder dysfunction. Standing, walking, and coughing might worsen the illness. Bed rest has been shown to ease pain, and the symptoms often appear intermittently [15]. Conventional radiography may not reveal pathological deviations but can reveal osseous erosion in the spinal canal or neural foramina [16]. CT scans can reveal cystic masses and bony alterations. MRI is the best method for investigating perineural cysts, as it provides heightened contrast for soft tissues and shows a hypointense signal on T1 and T2 weighted images [17]. A growing body of academic research has emerged, shedding light on compelling evidence indicating positive results in individuals who meet the diagnostic criteria for cervical radiculopathy when undergoing a comprehensive treatment plan. This plan includes diverse modalities such as manual therapy, mechanical traction, and precision-targeted strengthening exercises [18]. Research by Cleland et al. suggests that a multimodal intervention for cervical radiculopathy can predict positive outcomes. The study examined a therapist's approach, which included manual therapy techniques and targeted exercises, focusing on strengthening deep neck flexors and scapular stabilizers [19]. The absence of a positive cervical distraction test led to the exclusion of mechanical traction from the treatment regimen. Past and current studies also highlighted the effectiveness of thoracic thrust manipulation in enhancing cervical active range of motion and mitigating neck pain [20,21]. Neural mobilization techniques and soft tissue mobilization have gained prominence as integral treatments for cervical radiculopathy [22]. The patient showed significant improvements in elbow range of motion and pain reduction after soft tissue mobilization. The findings suggest its positive effect on the benefits of soft tissue mobilization in managing cervical radiculopathy [23].

## Conclusions

This case report delves into the effective management strategies employed for a patient presenting with cervical radiculopathy stemming from the presence of Tarlov cysts, a pathophysiological condition characterized by a myriad of neurological deficits, sensory abnormalities, and bladder dysfunction. In order to thoroughly comprehend the cystic formations, the patient was subjected to a series of radiological investigations, including CT scans and MRI. To address the multifaceted nature of the patient's condition, a comprehensive and integrative physiotherapeutic approach was meticulously implemented, with a primary focus on alleviating pain, improving neural mobility, and enhancing cervical spine function. The results of this case study were truly astounding, as the patient exhibited a remarkable response to the physiotherapy intervention, evidenced by a significant reduction in pain intensity and a noteworthy improvement in both cervical and upper extremity mobility.
